# *In situ* polarization and dielectric property measurements of Pb(Zr_0.52_Ti_0.48_)O_3_ ferroelectric nanocrystals

**DOI:** 10.1016/j.heliyon.2017.e00313

**Published:** 2017-06-07

**Authors:** Haifa Zhai, Yurong Jiang, Hongjing Li, Panpan Zhang, Yixiao He, Dandan Shi, Xiang Zhang, Jien Yang

**Affiliations:** a Henan Key Laboratory of Photovoltaic Materials, College of Physics and Materials Science, Henan Normal University, Xinxiang 453007, People’s Republic of China; b National Laboratory of Solid State Microstructures, Nanjing University, Nanjing 210093, People’s Republic of China

**Keywords:** Condensed matter physics, Nanotechnology, Materials science

## Abstract

Pb(Zr_0.52_Ti_0.48_)O_3_/polycarbonate (PZT/PC) composite films with different concentration of PZT ferroelectric nanocrystals are prepared. The polarization and dielectric relaxation behavior of PZT ferroelectric nanocrystals are characterized using *in situ* transmittance and X-ray diffraction (XRD) measurements for the first time. It’s found that 10% PZT/PC composite film has the largest orientation change and negligible dielectric relaxation after poling (the *φ* value of 13.8% is almost constant with time even for 168 h). Based on the XRD results, we consider that the preferential orientation of PZT nanocrystals to align in PC matrix after poling is [001] direction.

## 1 Introduction

Lead zirconate titanate [Pb(Zr_0.52_Ti_0.48_)O_3_, PZT] solid solution, one of the most popular ferroelectrics, has wide applications due to its excellent ferroelectric, pyroelectric, piezoelectric and electro-optic (EO) properties [1, 2, 3]. Remarkable spontaneous polarization (82 μC/cm^2^), high Curie temperature (450 °C) and huge piezoelectric coefficient *d*_33_ (31 PC/N) makes it a superior ferroelectric materials for multifunctional optoelectronic applications [4], such as optical modulators [5], optical switches [6] and optical waveguide devices [7]. Single crystal PZT films on various substrates exhibit large EO coefficient of ∼76 pm/V on Si wafer [8], 270.8 pm/V on Nb-SrTiO_3_ (001) wafer [9]. As an optimal candidate ferroelectric material for optoelectronic applications, it’s necessary to study the relationship between EO properties and polarization behavior so as to extend its application further.

For optical applications, single crystal materials of high structural perfection are optimal to obtain strong electro-optic effects, while the growth difficulties and high production cost hinder their commercial applications [10]. Thin films always require special substrates, such as SrTiO_3_ substrate [9, 10, 11]. The quality and crystallographic orientation of the films strongly influence the EO properties of thin films [12]. Besides, composites with ferroelectric nanocrystals as fillers have attracted much attention since the discovery of random lasers with extremely high nonlinear optical properties using ferroelectric powders [13]. Embedding the ferroelectric nanocrystals in a transparent matrix, such as optical organic polymers, is an effective method for the application in the field of electro-optic optics. Many kinds of organic polymers have been investigated for the optical applications, such as poly(methyl methacrylate) (PMMA) [14], polycarbonate (PC) [15] and polyimide (PI) [16]. As the polymer host, bisphenol A polymer polycarbonate ([-C_16_H_14_O_3_-]_n_) has many intrinsic advantages such as high transparency, low dielectric constant and loss, proper glass transition temperature (∼145 °C) and morphological plasticity. Low dielectric constant and loss are beneficial to ensure that the composite film has a high EO figure of merit [17].

The ferroelectric nanocrystal/polymer composites integrate the merits of ferroelectrics and polymers together to exhibit large EO figure of merit. At the same time, organic polymer-based composites exhibit many advantages such as flexibility, light weight and possible physicochemical property modulation by polymerization technique [18]. Pb(Zr_0.52_Ti_0.48_)O_3_/polycarbonate (PZT/PC) composite films combine both the advantages of PZT nanocrystals and PC polymers and exhibit good EO coefficient and figure of merit of 30.5 and 48.9 pm/V, respectively, much larger than those of industry standard LiNbO_3_ crystal, suggesting that PZT/PC composite film is a promising candidate for optoelectronic applications [13].

In the pristine ferroelectric nanocrystal/polymer composite films, the orientation distribution of ferroelectric nanocrystals is random, so it’s necessary to produce noncentrosymmetric order in composite films through poling. Corona-onset poling at elevated temperature (COPET) is always used to pole the composite films with homogeneous electric field distribution. The orientation distribution of ferroelectric nanocrystals plays the main role in E-O properties, so the characterization of the orientation distribution change of ferroelectric nanocrystals in polymer matrix is beneficial to understand the origin of EO properties enhancement. After poling, the composite films exhibit better E-O properties because of the orientation rearrangement of ferroelectric nanocrystals. The new distribution state can change with time, that is, the dielectric relaxation behavior occurs, which results in the degradation of E-O properties. So the studies of dielectric relaxation behavior and the stability of the optical device are essential, whereas there are fewer literatures [19]. In this paper, using PC as matrix, PZT/PC composite films were fabricated with different weight concentration of PZT nanocrystals. The polarization and dielectric relaxation behavior were investigated using *in situ* transmittance and X-ray diffraction (XRD) measurements for the first time, which also illustrated the preferential alignment orientation of PZT ferroelectric nanocrystals after poling.

## 2 Experimental

**Fig. 1:**
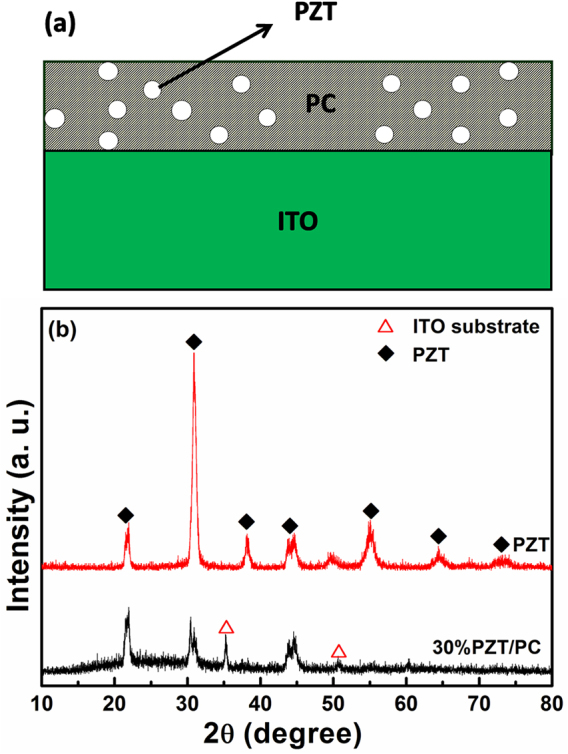
(a) Schematic diagram of the device structure of PZT/PC composite films; (b) XRD patterns of PZT nanocrystals and 30% PZT/PC composite film.

PZT ferroelectric nanocrystals were prepared by a hydrothermal method, as described detailedly in a literature using lead nitrate (Pb(NO_3_)_2_), oxyzirconium chloride (ZrOCl_2_ ·8H_2_O) and TiCl_4_ as starting chemicals [13]. The as-prepared nanopowders were dried at 100 °C for 12 h without further thermal treatment. Then different weight ratios of PZT nanocrystals were sonicated and dispersed into PC solution, chloroform used as solvent. The weight ratios of PZT to PC were 0, 10%, 20% and 30%, respectively. The suspension solutions were deposited on ITO glass substrates by the spin-coating method to obtain PZT/PC composite films, as illustrated in Fig. 1(a). The final thick films (∼1.2 μm) were baked at 60 °C in air to remove the excess solvent.

The XRD patterns of PZT nanocrystals and PZT/PC composite films were recorded on Rigaku-D/Max 2000 with Cu Kα radiation. A Shimadzu UV-3600 spectrometer was used to carry out the transmittance measurement. Corona poling techniques were used to pole the composite films with a high voltage of 8 kV and time period of 20 min at 155 °C. During the poling process, the location of top conducting tungsten needle was above the composite films with poling current of 0.6 μA and the ITO substrate was subjected as bottom electrodes. To investigate the polarization and dielectric relaxation behavior of PZT nanocrystals in PC matrix, *in situ* transmittance were carried out by introducing orientational order parameter (*φ*). *In situ* XRD measurement was used to analyze the preferential alignment orientation of PZT ferroelectric nanocrystals after poling; also revealed the dielectric relaxation behavior of composite films for the first time.

## 3 Results and discussion

The XRD patterns of PZT and 30% PZT/PC composite films are shown in Fig. 1(b). It’s clearly seen that the PZT ferroelectric nanocrystals prepared by the hydrothermal method are pure tetragonal perovskite phase (JPCDS card 33–0784). Typical peaks at 2*θ* = 43.6° and 44.8° assigned to (002) and (200) planes can be used to analyze the alignment orientation of PZT ferroelectric nanocrystals in next work. The XRD pattern of 30% PZT/PC composite film also displays in Fig. 1(b) with the main diffraction peaks of PZT nanocrystals the same.

For nonlinear optical (NLO) polymers, the rearrangement of the dipolar molecules always results in the decrease of adsorption intensity, compared with that before poling. The orientational order parameter (*φ*) is widely used to characterize the orientation change of chromophores before and after poling in EO polymers [20, 21], as described as follows:

(1) $$\phi =1-\frac{A_{⊥}}{A_{0}}$$

where A_0_ and A_⊥_ are the absorption peaks intensities of the film before and after poling [21]. By introducing the orientational order parameter, the polarization and dielectric relaxation behavior of PZT/PC are characterized with a modified equation due to the decrease of transmission after ferroelectric nanocrystals arrangement by poling. The Eq. (1) can be modified as follows:

(2) $$\phi =1-\frac{T_{⊥}}{T_{0}}$$

where T_0_ and T_⊥_ are the transmission valleys intensities of the film before and after poling.

**Fig. 2:**
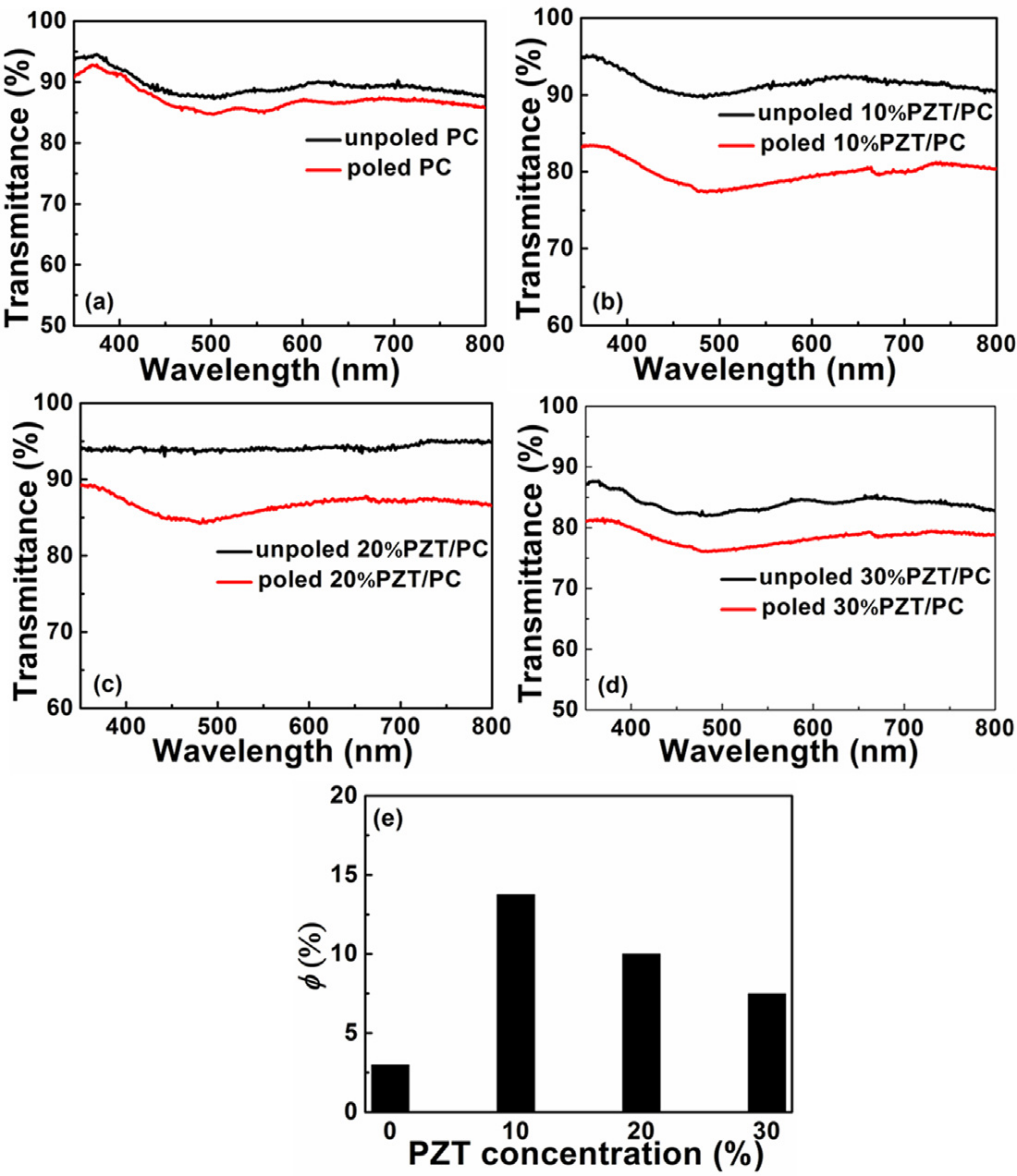
Transmittance spectra of PZT/PC composite films before and after poling with different PZT concentration of (a) 0, (b) 10%, (c) 20% and (d) 30%, respectively; and (e) the dependence of orientational order parameter (*φ*) on PZT concentration, calculated based on Eq. (2).

The transmittance spectra and orientational order parameters (*φ*) of PZT/PC composite films with different PZT concentration of 0, 10%, 20% and 30% are displayed as Fig. 2(a), (b), (c) and (d), respectively. As seen in Fig. 2, after poling, the transmittances of all composite films decrease due to the rearrangement of PZT nanocrystals under electric field, especially for the 10% PZT/PC composite films. In Fig. 2(e), the change of *φ* value with different PZT concentration shows that the weight ratio of 10% is the appropriate concentration with the largest *φ* value of 13.8%, which means the best second order NLO properties. The decrease of *φ* value with larger concentration of PZT can be attributed to the aggregation of PZT nanocrystals, which makes PZT nanocrystals difficult to change alignment orientation under electric field.

**Fig. 3:**
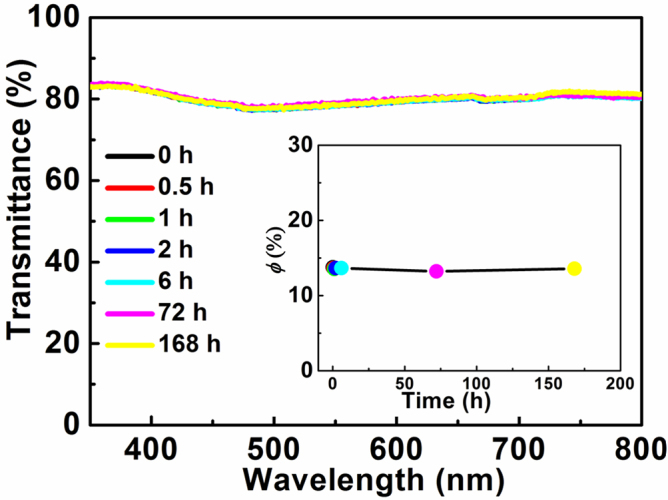
The temporal dependence of transmittance spectra and orientational order parameter (inset) of 10% PZT/PC composite film after poling at room temperature.

**Fig. 4:**
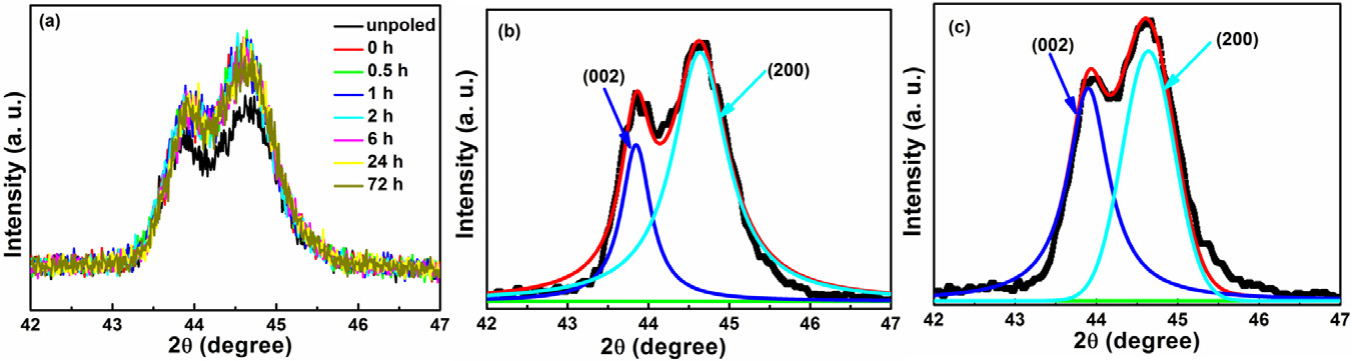
(a) The temporal dependence of XRD patterns and corresponding Lorenz fitting results of 10% PZT/PC composites films at 2*θ* = 42−47° (b) before and (c) after poling.

To investigate the stability of PZT nanocrystals in PC matrix, the dielectric relaxation behavior of 10% PZT/PC composite film is studied via *in situ* measurements, including *in situ* transmittance and XRD measurements, as shown in Fig. 3 and Fig. 4(a), respectively. Fig. 3 shows the temporal dependence of orientational order parameter of 10% PZT/PC composite films at room temperature, calculated based on Eq. (2). The *φ* value is almost constant with time, even after 168 h (a week), which means the polarization state is stable with negligible dielectric relaxation. That is to say, after poling, the orientation distribution of PZT nanocrystals changes to a new stable state. *In situ* XRD measurement of 10% PZT/PC also illustrates that the orientation of PZT nanocrystals changes after poling, while the new orientation can be kept stable even after 72 h, as seen in Fig. 4(a). It can be seen that after poling, the relative intensity of diffraction peaks in XRD patterns is almost the same with time. It also confirms that the dielectric relaxation is negligible for PZT nanocrystals in PC matrix after poling. For Dispersed Red 1 (DR1) doped guest-host polymers, such as DR1/PC, DR1/PMMA and DR1/poly(2-vinylpyridine)(P2VP), the normalized order parameters decrease to 86%, 69% and 50% of its original value after several days [22, 23]. Compared with chromophores doped E-O composites, PZT/PC composite films after poling are stable, with negligible dielectric relaxation.

From the results above, it can be concluded that the PZT nanocrytals rearrange to a new orientation distribution state after poling, while the preferential orientation of PZT nanocrystals to align in PC matrix is still not clear. For tetragonal PZT films, the favorable spontaneous polarization is parallel to [001] direction [24, 25], which means it’s the most susceptible direction under external electric field and has the optimal E-O properties. It shows that (001)-oriented PZT thin film has the largest linear EO coefficient compared to (011) and (111)-oriented thin films [9]. In order to illuminate the preferential orientation, the XRD patterns and corresponding Lorenz fitting results of 10% PZT/PC composites at 2*θ* = 42−47° before and after poling are shown in Fig. 4(b) and (c), respectively. Based on the assumption, the ratio of [001] orientation of PZT nanocrystals should be increased after poling, which means the intensity ratio of (001) plane increase in XRD patterns, same as (002) plane [19]. In bulk and film samples, the intensity ratio of diffraction peaks is always used to roughly estimate the preferential orientation of nanocrystals under certain conditions [26]. In Fig. 4(b) and (c), the intensity ratio of (002)/(200) diffraction peaks change from 62.8% to 85.2%, which means the orientation of some PZT nanocrystals change to [001] direction from random distribution after poling and the electrical domain change in PZT nanocrystals may also contribute the change. So we think the preferential orientation of PZT nanocrystals to align in PC matrix is [001] direction.

## 4 Conclusions

PZT/PC composite films with different concentration of PZT nanocrystals are prepared by the spin coating method. The polarization and dielectric relaxation behavior of PZT/PC composite films are characterized using *in situ* transmittance and XRD measurements for the first time. It’s found that 10% PZT/PC composite film has the largest orientation change and negligible dielectric relaxation after poling (the *φ* value of 13.8% is almost constant with time even for 168 h). *In situ* XRD measurement of 10% PZT/PC also illustrates that the orientation of PZT nanocrystals changes to a new stable orientation distribution state after poling. Based on the XRD results, we consider that the preferential orientation of PZT nanocrystals to align in PC matrix after poling is [001] direction.

## Declarations

### Author contribution statement

Haifa Zhai: Conceived and designed the experiments; Performed the experiments; Analyzed and interpreted the data; Wrote the paper.

Yurong Jiang: Analyzed and interpreted the data; Contributed reagents, materials, analysis tools or data.

Hongjing Li, Panpan Zhang, Yixiao He, Dandan Shi and Xiang Zhang: Performed the experiments.

Jien Yang: Analyzed and interpreted the data.

### Funding statement

This work was supported by the Natural Science Foundation of China (No. 51202107), a grant from the Opening Funding of National Laboratory of Solid State Microstructure (No. M26017) and Foundation of Henan Educational Committee (No. 16A140028).

### Competing interest statement

The authors declare no conflict of interest.

### Additional information

No additional information is available for this paper.
